# The benefit of vegetarian diets for reducing blood pressure in Taiwan: a historically prospective cohort study

**DOI:** 10.1186/s41043-023-00377-3

**Published:** 2023-05-09

**Authors:** Hsin-Pei Feng, Pi-Ching Yu, Shi-Hao Huang, Yao-Ching Huang, Chin Fu Chen, Chien-An Sun, Bill-Long Wang, Wu-Chien Chien, Chun-Hsien Chiang

**Affiliations:** 1 School of Nursing, National Defense Medical Center, Taipei City, 11490 Taiwan; 2 Cardiovascular Intensive Care Unit, Department of Critical Care Medicine, Far-Eastern Memorial Hospital, New Taipei City, 10602 Taiwan; 3 Graduate Institute of Medical Sciences, National Defense Medical Center, Taipei, 11490 Taiwan; 4 Department of Medical Research, Tri-Service General Hospital, Taipei, 11490 Taiwan; 5 School of Public Health, National Defense Medical Center, Taipei, 11490 Taiwan; 6 Department of Chemical Engineering and Biotechnology, National Taipei University of Technology (Taipei Tech), Taipei, 10608 Taiwan; 7 Amed Advanced Medication Co., Ltd., New Taipei City, 24890 Taiwan; 8 Center for Technology Transfer and Resources Integration, Fu-Jen Catholic University, New Taipei City, 242062 Taiwan; 9 Department of Public Health, College of Medicine, Fu-Jen Catholic University, New Taipei City, 242062 Taiwan; 10 Big Data Center, College of Medicine, Fu-Jen Catholic University, New Taipei City, 242062 Taiwan; 11 Taiwanese Injury Prevention and Safety Promotion Association (TIPSPA), Taipei, 11490 Taiwan; 12 Graduate Institute of Life Sciences, National Defense Medical Center, Taipei, 11490 Taiwan; 13 Department of Cardiovascular Medicine, Far-Eastern Memorial Hospital, New Taipei City, 10602 Taiwan

**Keywords:** Diastolic blood pressure, Hypertension, Systolic blood pressure, Vegetarian

## Abstract

**Objective:**

Past vegetarians research has often found that they have lower blood pressure (BP). Effects may include their lower BMI and higher intake levels of fruit and vegetables. Besides, the study pursues to extend this evidence in a diverse population containing vegans, lacto-ovo vegetarians and omnivores.

**Design:**

The study analyzed data on five hundred vigorous individuals aged 20 years or older from a standard medical screening program and provided validated questionnaire. Criteria were established for vegan, lacto-ovo vegetarian, partial vegetarian and omnivorous dietary patterns.

**Setting:**

Health screening programs were conducted at a standard medical screening program in Taiwan between 2006 and 2017. Dietary data were gathered by self-administered questionnaire.

**Subjects:**

Five hundred Taiwanese subjects representing the cohort.

**Results:**

Multiple regression analyses confirmed that the vegan vegetarians had lower systolic and diastolic BP (mmHg) than omnivorous Taiwanese (β =  − 6.8, *p* < 0.05 and β =  − 6.9, *p* < 0.001). Findings for lacto-ovo vegetarians (β =  − 9.1, *p* < 0.001 and β =  − 5.8, *p* < 0.001) were similar. The vegetarians were also less likely to be using antihypertensive medications. Defining hypertension as systolic BP > 139 mmHg or diastolic BP > 89 mmHg or routine of antihypertensive medications, the odds ratio of hypertension compared with omnivores was 0.37 (95% CI = 0.19–0.74), 0.57 (95% CI = 0.36–0.92) and 0.92 (95% CI = 0.50–1.70), respectively, for vegans, lacto-ovo vegetarians and partial vegetarians. Results were reduced after adjustment for BMI.

**Conclusions:**

The study concludes from this relatively large study that vegetarians, especially vegans, with otherwise diverse characteristics but stable diets, do have lower systolic and diastolic BP and less hypertension than omnivores.

## Introduction

Delighted blood pressure (BP) is a changeable major risk factor for cardiovascular morbidity and mortality. Estimates are that suboptimal BP (systolic BP > 115 mmHg) is responsible for 62% of cerebrovascular diseases and 49% of IHD, with little variation by gender [1]. National Center for Health Statistics Health estimates an overall 31.3% prevalence rate of hypertension (systolic BP ≥ 140 mmHg or diastolic BP ≥ 90 mmHg or taking medications for hypertension) in 2003–2006 [2], and of 47.3% in those aged 55 years or older [3]. This suggests that fifty million or more Americans have higher BP. Propitious BP levels are associated with greater probability of survival to age 85 years as well as increased longer life without comorbidity [4]. The prevalence of hypertension increases substantially with age and that the elderly population is growing rapidly. However, the management of hypertension population deserves significant attention. Eduardo and his colleagues [5] discuss the common problems associated with the diagnosis and management of hypertension in the elderly, including the phenomena of ‘pseudo-hypertension’ and postural and post-prandial hypotension, and the high likelihood of comorbidity and mortality [6]. They also highlight critical reflections when deciding on the antihypertensive treatment form to employ, and the latest research on how aggressively to control BP in these patients.

The differences prevalence rates of hypertension between definite populations and age groups have been partially explained by differences in intakes of particular nutrients, although the evidence for some is weak. These include positive associations with Na, alcohol and total protein, and negative associations with K, Ca and Mg [5, 6]. The Dietary Approaches to Stop Hypertension (DASH) diet research recently shows superior to a more typical diet but containing more lean red meat [7] in post-menopausal women, However, results of feeding studies of meat on BP are inconsistent [8, 9]. Dietary fats have been studied, with some studies showing no convincing effects on BP, while other studies indicate a possible relationship depending on fat type [10–15]. Fiber has also been studied, and recent meta-analyses of trials have shown small but significant effects mainly on diastolic BP and more pronounced effects in hypertensive individuals [16, 17].

The results of a vegetarian diet on BP have been evaluated by several previous studies over past thirty years. Most of these have been small short-term feeding studies [9, 18–20] or cross-sectional comparisons also of fairly small, selected study groups of vegetarians and others [21–24]. Melby et al. have studied this association in African Americans [25, 26] with mixed results. Most of these studied the lacto-ovo vegetarian rather than vegan diet. An omission to limited size and diversity of subjects is a very large cross-sectional comparison of British vegetarians (vegans and lacto-ovo vegetarians) and health-conscious non-vegetarians [27], where significant impacts were found, mainly in the vegans. In the current study we display results of a cross-sectional comparison within a relatively large and diverse group where the vegetarian and non-vegetarian habits were generally long term, and where vegetarians were divided to vegans and non-vegans. This study group is representative of a national cohort of healthy individuals aged 20 years or older from a standard medical screening program [28] which spans a wide range of socio-economic status, age and geographic location, and both genders.

## Materials and methods

### Data sources

The MJ Health Data Database is a long-term tracking, large-scale, and comprehensive population health database. Since 1994, the health database has collected data on participants who received health examination services at the MJ Health Management Agency. In addition to health data, some participants agreed to donate blood samples as early as 2002. MJ Health Management has four health examination centers in Taiwan, located in Taipei (Northern Region), Taoyuan (Northwest Region), Taichung (Central Region), and Kaohsiung (Southern Region), providing comprehensive services to people in Taiwan and neighboring regions in Asia. The health database stores the actual data of the participants’ previous physical health examination findings. The data can be traced back to the health examination results in 1994. Therefore, many participants in the health database have multiple health examination records. Information, such as type, is collected through health questionnaires, and the human body and biochemical test data are collected through health examinations. These data are continuously collected with no expiration date.

### Study design

The study analyzed 500 healthy individuals aged 20 years or older who participated in a standard medical screening program run by the MJ Health Management institution in (Taipei, Taiwan. All participants were followed up between 2006 to 2017. For each person, a self-administered questionnaire collects information on demographic characteristics, lifestyle and health risk. During hands-on physical examinations by physicians, medical histories were taken. Blood pressure measurement was performed with a mercury sphygmomanometer by well trained professional nurses and was the average of two readings in a sitting position for the left arm. The process was standardized to minimize the intra- and inter- observer variability.

Hypertension is defined as those with systolic blood pressure $\ge$ 140 mmHg at the time of initial screening or those with a history of hypertension or taking hypertension medications [28]. Relevant data were gathered from a self-administered FFQ and a 7- day physical activity recall. Study subjects came to the clinic fasting, having been educated to take their usual medications. They were instructed to empty their bladder and were then seated in a quiet room at a comfortable temperature for approximately 10 min before their systolic and diastolic BP were measured using an Omron automated sphygmomanometer [30]. Three readings were taken 5 min separately. The mean of the second and third readings was used for the Pettersen et al. Present analyses unless there was a difference of more than 5 mmHg, in which case we used the mean of all three readings. We let off four subjects with a mean recorded systolic BP of less than 80 mmHg, leaving 500 subjects for analysis.

Dietary pattern was determined using information from the FFQ about intake of twenty-five different food items (Table 1) relevant to vegetarian status.

**Table 1:** The twenty-five items from the FFQ used for classification of dietary patterns

Red meat (3 items)
Hamburger, ground beef
Processed beef, lamb
Beef or lamb a main dish
Poultry (2 items)
Processed chicken or turkey
Chicken or turkey
Fish (4 items)
White fish
Salmon
Canned tuna, tuna salad, etc
Other fish
Dairy products (15 items)
Cottage cheese
Cream cheese, cheese spread
American processed, cheddar cheese
Low-fat cheese, mozzarella, ricotta
Butter
Milk (whole or 2%)
Low-fat milk
Evaporated milk
Low-fat yoghurt
Regular yoghurt
Other dairy products
Ice cream, milk shakes
Ice milk, frozen yoghurt
Meal replacement drink
Hot chocolate
Eggs (1 item)

Participants were asked to report their usual or average diet during the past year. Each food item allowed up to nine frequency response options. The validity of this FFQ is relatively high for most nutrients and foods when compared with the means of the six dietary recalls [31, 32]. Frequencies of intake were converted to daily equivalents, and these were used to construct composite food variables that measured intakes of red meat, poultry, fish, eggs and dairy foods. Values of these variables allowed subjects to be allocated to a dietary pattern as shown in Table 2.

**Table 2:** Definitions of dietary patterns

Main dietary pattern	Definition
Vegan	Eat meat, fish and dairy less than once monthly
Lacto-ovo vegetarian	Eat meat and/or fish less than once monthly, and dairy more than once monthly
Partial vegetarian	Includes pesco-vegetarians, who eat meat less than once monthly and fish at least once monthly, and semi-vegetarians, who eat meat at least once monthly and fish and meat less than once weekly
Non-vegetarians	No specific dietary restrictions as to frequencies of meat, fish and dairy

There were small amounts of missing data in the dietary variables. For about half of these variables a random 10% subset of initially missing data was later filled-in by telephone. We used this to guide imputation [33] of the remaining missing data for these variables. For other variables we presumed that the data were missing at random, which even if not quite correct will cause little bias when the missing data rate is small [33]. Imputation was performed at the level of the composite variables’ meat, fish, dairy and eggs, and was also conditional on other covariates and the dependent variable in any particular statistical model. The imputation software used was the SPSS 22.0 version [34]. Physical activity was measured using a detailed hour-by-hour telephone recall about type, intensity and duration of different physical activities during the preceding week [35]. These activities were summed to produce hours per week of moderate, hard or very hard exercise (metabolic equivalent task levels ≥ 4.5). Information on medication use in the present study was obtained along with the 24 h recalls. A cardiologist subsequently identified all medications used for treatment of or known to reduce BP. The use of such medications is reported as none or some. This population largely abstains from alcohol use (6.75% admit to current use, mostly infrequent), although it was tested as a covariate. The study was conducted in accordance with the Declaration of Helsinki and all subjects gave written informed consent. The study was approved by the Institutional Review Board of Tri-Service General Hospital (TSGHIRB No.: B-109-30).

### Statistical analysis

MANOVA was performed to test null hypotheses of equal means between dietary groups for continuous variables, while the χ2 test was used for categorical variables. These were performed using the SPSS statistical software package release 22.0 (SPSS Inc., USA). Linear and logistic regression modeling was conducted by using statistical software. Where required hypotheses involving interactions were tested using product terms in regression models.

## Results

### Demographics

The four groups of participants are presented in Table 3. Ten per cent were vegan, 36% were lacto-ovo vegetarian, 14% partial vegetarian and 40% non-vegetarian (Table 3).

**Table 3:** Main characteristics of the study population: subjects representing the Taiwanese health screening program cohort, Taiwan

Variable	Vegan (*n* = 49; 10%)		Lacto-ovo vegetarian (*n* = 184; 36%)		Partial vegetarian (*n* = 69; 14%)		Non-vegetarian (*n* = 198; 40%)		All (*n* = 500)
	*n* or mean	% or SD	*n* or mean	% or SD	*n* or mean	% or SD	*n* or mean	% or SD	*p*-value
*Age (years)*									
20–39	0	0	5	3	3	4	8	4	0.03^**∥**^
40–49	1	2	33	18	11	16	43	22	
50–59	13	27	46	25	18	26	44	22	
60–69	13	27	33	18	21	31	48	24	
≥ 70	22	44	67	36	16	23	55	28	
*Mean and sd*	67·6	11·6	63·5	14·9	61·4	12·6	61·0	13·2	0·01^**¶**^
Gender									
Women	35	71	116	63	47	68	122	62	0.52^**∥**^
*Education*									
High school or below	16	33	34	19	14	21	39	20	0·28^**∥**^
Some college	16	33	68	38	22	33	82	43	
Bachelors and above	16	33	77	43	30	46	70	37	
*BMI*^**†**^ (kg/m^2^)									
≤ 24·9	35	72	109	59	32	46	53	27	< 0·0001^**∥**^
25·0–29·9	8	16	52	28	22	32	69	35	
≥ 30·0	6	12	23	13	15	22	76	38	
Mean andsd	24·0	5·9	25·1	5·2	26·3	5·2	29·5	6·6	< 0·0001^**¶**^
*BP*^**‡**^ (range) (mmHg)									
All	(*n* 49)		(*n* 184)		(*n* 69)		(*n* 198)		
Systolic (83·0–199·0), mean andsd	123·5	21·6	123·2	20·8	124·4	19·5	127·9	18·2	0·11^**¶**^
Diastolic (48·5–109·0), mean andsd	71·3	8·8	73·0	9·6	75·4	9·6	78·1	9·6	< 0·0001^**¶**^
Untreated	(*n* 44)		(*n* 139)		(*n* 50)		(*n* 141)		
Systolic (83·0–184·5), mean andsd	123·0	22·4	119·3	19·1	121·1	19·1	126·1	17·3	0·03^**¶**^
Diastolic (48·5–109·0), mean andsd	70·9	9·1	72·6	19·1	74·2	8·6	78·3	9·7	< 0·0001^**¶**^
Treated	(*n* 5)		(*n* 45)		(*n* 19)		(*n* 57)		
Systolic (90·3–199·0), mean andsd	127·6	12·1	135·4	21·3	132·9	18·2	132·4	19·8	0·80^**¶**^
Diastolic (54·5–100·5), mean andsd	74·7	5·2	74·0	9·5	78·5	11·6	77·5	9·1	0·21^**¶**^
*BP medication*									
No	44	90	139	76	50	72	141	71	0·06^**∥**^
Some	5	10	45	24	19	25	57	29	
Women	3	60	26	58	12	63	32	56	0·96^**∥**^
Men	2	40	19	42	7	37	25	44	
*Duration of membership*^**§**^									
< 25 years	6	13	14	8	11	16	39	21	0·0044^**∥**^
25–50 years	10	21	51	29	27	40	63	33	
50–75 years	23	49	92	51	24	36	70	37	
≥ 75 years	8	17	22	12	5	8	16	9	

*BP* Blood pressure

Data are presented as numbers and percentages or means and standard deviations where appropriate, with significance tests (*n* = 500)

^†^13 missing BMI data

^‡^Mean of last two BP measurements if difference between last two measurements is ≤ 5 mmHg; mean of three BP measurements if difference between last two measurements is > 5 mmHg

^§^19 missing data on age

^∥^*P* value for *χ*^2^ test for dependency among dietary patterns

^¶^*P* value for ANOVA test of equality of means among dietary patterns

The age distribution was substantially different between the groups with the vegans being older. The average age of the participants was 62.7 years and 64% were women. There was a large range of educational attainment and subjects lived in all major regions of Taiwan.

### Blood pressure and hypertension medication

The average BP levels were relatively low for all subjects (systolic 125.2 mmHg, diastolic 75.2 mmHg), moderately higher for those treated (systolic 133.3 mmHg, diastolic 76.3 mmHg) and lowest in untreated subjects (systolic 122.5 mmHg, diastolic 74.8 mmHg). The proportion taking some medication known to reduce BP was for the entire population 25.2%; 22.8% for women and 29.4% for men. The study found no significant differences in BP between the four dietary groups among those taking anti-hypertensive medication.

### Blood pressure, age, gender and physical activity

There were no significant differences in BP by gender. As expected, there were strong age effects on systolic BP, but these were not seen for diastolic BP. The physical activity could not demonstrate significant effects on BP.

### Relationship between diet and blood pressure

Adjusting for age and gender risk factors (Table 4) and including only non-treated individuals, systolic BP was significantly lower in vegans and lacto-ovo vegetarians (*β* =  − 6.8 mmHg, *p* < 0.05 and *β* =  − 9.1 mmHg, *p* < 0.001) when compared with non-vegetarians, and results were broadly similar for diastolic BP (*β* =  − 6.9 mmHg, *p* < 0.001 and *β* =  − 5.8 mmHg, *p* < 0.001; Table 4). There was a significantly higher systolic BP at older ages (0.6 mmHg higher per year, *p* < 0.001) and there was no distinct effect of physical activity.

**Table 4:** Parameter estimates (*β* coefficient, 95% confidence interval) relating blood pressure (BP) and dietary pattern by antihypertensive medication status, adjusted for gender and age: subjects representing the Taiwanese Health Study

Variable	Systolic BP				Diastolic BP			
	No treatment (*n* 374)		All (*n* 500)		No treatment (*n* 374)		All (*n* 500)	
	*β*	95% CI	*β*	95% CI	*β*	95% CI	*β*	95% CI
Non-vegetarian	0·0		0·0		0·0		0·0	
Partial vegetarian	− 5·1	− 10·6, 0·4	− 4·9	− 10·5, 0·8	− 4·4^**^	− 7·5, − 1·2	− 4·2^**^	− 7·3, − 1·07
Lacto-ovo vegetarian	− 9·1^***^	− 13·1, − 4·9	− 8·9^***^	− 13·1, − 4·7	− 5·8^***^	− 8·1, − 3·4	− 5·7^***^	− 7·5, − 2·9
Vegan	− 6·8^*^	− 12·7, − 1·0	− 7·1^**^	− 12·9, − 1·4	− 6·9^***^	− 10·1, − 3·7	− 6·6^***^	− 9·0, − 3·1
Gender (female/male)	0·7	− 3·0, 4·5	− ·5	− 0·8, 5·9	− 1·2	− 3·3, 1·0	− 0·8	− 2·6, 1·0
Age (per year)	0·6^***^	0·5, 0·8	0·6^***^	0·5, 0·8	− 0·003	− 0·08, 0·08	− 0·04	− 0·1, 0·02
Medication (*n*o/yes)	No entries		0·6	− 4·8, 6·1	No entries		0·57	− 3·5, 2·4
Exercise^**†**^	− 0·01	− 0·3, 0·3	0·04	− 0·2, 0·3	− 0·05	− 0·2, 0·1	− 0·03	− 0·2, 0·1
Lacto-ovo × medication	No entries		9·5^*^	1·5, 17·6	No entries		No entries	

^*^p, 0·05 ^**^p,0·01 ^***^p,0·001

^†^Hours per week of moderate/hard/very hard physical activity

BMI was significantly associated with both systolic and diastolic BP (Table 5). Where normal BMI was the reference, overweight subjects (BMI = 25.0–29.9 kg/*m*^*2*^) had systolic BP 3.1 mmHg higher (NS) and obese subjects (BMI ≥ 30.0 kg/*m*^*2*^) on average had systolic BP 11.9 mmHg higher (*p* < 0.001). For diastolic BP these statistics were respectively 3.5 mmHg (*p* < 0.01) and 9.6 mmHg (*p* < 0.001; Table 5). Creating 2-unit wide BMI categories between BMI of < 18 kg/*m*^*2*^ and > 32 kg/*m*^*2*^ revealed a direct association with BP from lowest to highest categories for both systolic BP and diastolic BP.

**Table 5:** Parameter estimates (*β* coefficient, 95% confidence interval) relating blood pressure (BP) and dietary pattern by antihypertensive medication status, adjusted for BMI, age and gender: Taiwanese subjects representing the health screening cohort, Taiwan

Variable	Systolic BP				Diastolic BP			
	No treatment (*n* 374)		All (*n* 500)		No treatment (*n* 374)		All (*n* 500)	
	*β*	95% CI	*β*	95% CI	*β*	95% CI	*β*	95% CI
Non-vegetarian	0·0		0·0		0·0		0·0	
Partial vegetarian	− 2·8	− 8·3, 2·6	− 3·4	− 6·4, 3·0	− 2·6	− 5·5, 0·4	− 2·7	− 5·6, 0·2
Lacto-ovo vegetarian	− 5·9^**^	− 9·7, 21·5	− 6·0^**^	− 9·4, − 1·2	− 3·2^**^	− 5·4, 20·9	− 3·0^**^	− 5·3, 20·8
Vegan	− 3·8	− 10·7, 1·2	− 4·3	− 11·1, 0·2	− 4·2^**^	− 7·9, 21·4	− 4·3^*^	− 7·3, 20·3
BMI (kg/m^2^)								
≤ 24·9	0·0		0·0		0·0		0·0	
25·0–29·9	3·1	− 1·1, 7·0	− ·9	− 0·8, 6·5	3·5^**^	1·3, 5·6	3·8^***^	1·9, 5·7
≥ 30·0	11·9^***^	7·1, 16·4	10·8^***^	6·8, 14·9	9·6^***^	7·1, 12·1	9·5^***^	7·4, 11·6
Gender (female/male)	0·7	− 3·0, 4·3	− ·5	− 0·8, 4·0	− 1·3	− 3·3, 0·7	− 0·6	− 2·4, 1·1
Age (per year)	0·7^***^	0·5, 0·8	0·7^***^	0·5, 0·8	0·003	− 0·07, 0·07	− 0·02	− 0·1, 0·04
Medication (no/yes)	No entries		− 1·4	− 6·8, 4·0	No entries		− 2·3	− 2·1, 0·5
Exercise†	0·1	− 0·2, 0·4	0·1	− 0·1, 0·4	0·004	− 0·1, 0·1	0·01	− 0·1, 0·1
Lacto-ovo × medication	No entries		10·3^*^	− ·3, 18·3	No entries		No entries	

^*^p, 0·05; *^*^p,0·01; *^**^p,0·001

^†^Hours per week of moderate/hard/very hard physical activity

Adjusting for BMI the same trends with dietary pattern observed in Table 4, the differences were less (Table 5). Evidently BMI is to some extent an intermediary between diet and BP effect. However, significant dietary effects were still seen when the analyses were confined to the 224 subjects with BMI < 25.0 kg/*m*^*2*^. Definitely, with nonvegetarians as the reference, vegans had lower systolic BP by 7.12 mmHg (*p* = 0.06), lactoovo vegetarians were lower by 5.55 mmHg (*p* = 0.06) and partial vegetarians by 2.75 mmHg (*p* = 0.47). For diastolic BP vegans were lower by 5.10 mmHg (*p* = 0.006), lacto-ovo vegetarians were lower by 3.07 mmHg (*p* = 0.03) and partial vegetarians lower by 0.52 mmHg (*p* = 0.78). When adjusting for BMI these effects in non-overweight vegetarians diminished by 1–2 mmHg for systolic BP (test for dietary effects: *p* = 0.17 for vegans, *p* = 0.11 for lacto-ovo vegetarians) and by about 1 mmHg for diastolic BP (test for dietary effects: *p* = 0.02 for vegans, *p* = 0.06 lacto-ovo vegetarians).

Describing hypertension as average systolic BP > 139 mmHg or average diastolic BP > 89 mmHg or taking prescribed antihypertensive medications, logistic analysis showed that the vegetarian categories related to hypertension in a similar manner to that reported for BP.

(Table 6). Specifically, vegans, lacto-ovo vegetarians and partial vegetarians had lower assessed odds of hypertension (OR = 0.37 (95% CI = 0.19–0.74), OR = 0.57 (95% CI = 0.36–0.92) and OR = 0.92 (95% CI = 0.50–1.70)) than non-vegetarians and the odds ratios reduced significantly (OR = 0.53 (95% CI = 0.25–1.11), OR = 0.86 (95% CI = 0.51–1.45) and OR = 1.22 (95% CI = 0.64–2.33)) when the model added BMI. The effect of diet to reduce BP is partly mediated by dietary effects on BMI.

**Table 6:** Odds ratio and 95% confidence interval for hypertension- by dietary pattern with and without BMI: subjects representing the Taiwanese Health Study cohort, Taiwan (*n* = 500)

	OR estimates without BMI			OR estimates with BMI		
Effect	Point estimate	95% CI	*p*-value	Point estimate	95% CI	*p*-value
Non-vegetarian	0·00			0·00		
Partial vegetarian	0·92	0·50, 1·70	0·79	1·22	0·64, 2·33	0·55
Lacto-ovo vegetarian	0·57	0·36, 0·92	0·02	0·86	0·51, 1·45	0·57
Vegan	0·37	0·19, 0·74	0·005	0·53	0·25, 1·11	0·09
Gender	0·97	0·63, 1·50	0·90	0·99	0·63, 1·55	0·97
Age	1·07	1·05, 1·09	< 0·0001	1·08	1·06, 1·10	< 0·0001
Exercise	0·99	0·96, 1·02	0·50	1.00	0·96, 1·03	0·89
BMI (kg/m^2^)						
≤ 24·9				0·00		
25·0–29·9				1·53	0·92, 2·53	0·09
≥ 30·0				4·64	2·66, 8.11	< 0·0001

^†^Hypertension defined as average systolic blood pressure > 139 mmHg or average diastolic blood pressure > 89 mmHg or taking antihypertensive medications

Inserting alcohol intake to any of the models changed results only irrelevantly and the alcohol effect was always far from statistical significance. This is not surprising given the infrequent and small intakes.

In this cohort, overnight urinary K (but not Na or Ca) elimination correlates well with the corresponding dietary intake (*r* = 0.55 for K compared with six dietary recalls). Urinary K results were available from a random thirty-six calibration study subjects who were not taking antihypertensive medications. The latter were excluded as frequent use of diuretics will distort results. The overnight K excretion in vegetarians (vegans and lacto-ovo vegetarians combined) was on average 30.0 mmol/l and 19.8 mmol/l for non-vegetarians (*p* = 0.10). This suggests a substantial difference in K intake as would be expected.

## Discussion

This study presents significant differences in both systolic and diastolic BP and the odds of hypertension, depending on vegetarian dietary pattern among Taiwanese population. These results are from a population that includes a wide variety of age, socio-economic status and both genders, and they represent dietary habits stable over many years for the most part. Subjects summarized their diets over the previous year. Few other studies have been able to compare habitual non-vegetarians with both habitual vegans and lacto-ovo vegetarians. The impact seems to be moderately stronger in vegans as there were fewer vegans taking anti-hypertensive medications and those not taking such medications had BP as low (approximately) as the lacto-ovo vegetarians. Dietary effect appear partially explained on BMI, which are strong in this study [36]. Dietary effects were still marked in those with normal BMI.

The percentage overweight or obese in this study population was lower than in the general study population [36], though still considerable. These results are adjusted for those factors that the effect of a vegetarian diet to reduce body weight is one mechanism partially responsible for the BP effect. However, some additional effects probably still remain. A partial mediating effect of BMI is consistent with the results of some other observational studies [21, 27]. Remarkably, in previous short-term feeding studies there was typically no weight loss during the vegetarian feeding period, despite the well-known long term large differences in BMI between vegetarians and others. It does seem possible that no weight changes were seen over a few weeks of the feeding studies, physiological processes (e.g., insulin/glucose metabolism) [19] resulting in or associated with weight loss over a longer period may have begun and may already have started to affect BP.

Almost other similar studies, the study found effects on both systolic and diastolic BP. While a few related studies found changes in systolic [9, 20, 22] pressures only, this may have been due to limited statistical power.

Beyond BMI, which dietary factors in the vegetarian diet may account for effects on BP levels is not well understood. Vegetarians have higher fiber and K intakes as a result of their greater intakes of fruits, vegetables, fruits, nuts and wholegrain products [37–39]. Current meta-analyses of randomized trials [16, 17] demonstrate small but significant effects of Fiber, particularly in those with higher baseline pressures.

Consumption of a K-rich diet has a natriuretic effect and diets that are high in K usually are low in Na, as long as unprocessed foods are consumed [5]. Low Ca or dairy intake has also been associated with higher BP [40–42]. Inconsistent with this, our results and those from the European Perspective Investigation into Cancer and Nutrition (EPIC)–Oxford study [27] show vegans who avoid dairy products have the least hypertension. However, vegans do not have particularly low Ca intakes, perhaps due in part to supplementation but also because of vegetable sources of Ca. Proposed physiological mechanisms that may mediate the effect of a vegetarian diet include modulation of baroreceptor sensitivity, direct vasodilatory effects, changes in catecholamine and renin–angiotensin–aldosterone metabolism, improvement of glucose tolerance with lower insulin levels [5, 19], and lower blood viscosity in vegetarians [43].

The study measured BP using an automated sphygmomanometer which provides BP with acceptable validity [30]. A large amount of seemingly random error is associated with BP measurement, despite taking measures to neutralize the known influencing factors. In addition to using the digital machine we standardized other environmental factors that may influence BP. The relatively large number of studies subjects these factors should reduce the effects of random errors. Most study subjects had been church members for decades, further suggesting stability of dietary habits. Differences in duration of membership by dietary category seem unlikely to affect results given that only 8–21% had been members for < 25 years across the dietary groups. It was indispensable to assign vegetarian dietary pattern based on the results of an FFQ rather than the repeated recalls, as a small number of recalls will easily miss less frequent consumption of animal products. There is inevitably recall and reporting error in these data.

However, compared with the average of six 24 h recalls, correlation coefficients (*r*) corrected are as follows: red meat (*r* = 0.76); poultry (*r* = 0.76); fish (*r* = 0.53); eggs (*r* = 0.64); dairy protein (*r* = 0.77). Thus, by usual standards the validity of food frequency questions used in the algorithm assigning vegetarian status is excellent. Inevitably there were small amounts of missing dietary data (< 1% for most composite variables in this study). However, 9.4% of subjects were missing at least one of the longer lists of individual dairy items where we needed to assume missing at random for imputation and this may have resulted in a small amount of misclassification.

The proportion of this study population who were taking anti-hypertensive medication was 25.3%, compared with 21.3% in the United States population aged 18 years and above [44]. It is interesting that 10.0% of vegans but 28.8% of non-vegetarians are taking anti-hypertensive medications. This could mean that the vegan population is less willing to take medications or that BP is indeed lower in this subgroup. Given the relatively low BP among vegans not taking antihypertensive medications, their need for medications is probably less. The much lower odds of hypertension in this group are thus due both to the lower proportion taking antihypertensive medication and the low BP in those not taking medication.

The cross-sectional study do not know the stability of dietary patterns over time. Itis not possible to exclude a reverse causation in that some may have changed their diet after they received a diagnosis of elevated BP. However, if this occurred it would likely work against reverse causation as most Adventists with a health problem would actually move towards a plant-based diet.

## Conclusion

The study outspreads and supports previous evidence that diet affects measured BP levels, both systolic and diastolic, with vegans and lacto-ovo vegetarians having lower BP than nonvegetarians. The study shows that this appears to be long-lasting as study subjects generally have maintained these dietary habits over at least 1 year. The vegans appear to have the least hypertension, although further evidence should be gathered on this group. The study population represent a diverse population (although all Taiwanese) by gender, BMI, socio-economic status. Many peoples may benefit from a diet containing more plant foods to prevent hypertension.

## Data Availability

Data are available from the MJ Health Examination Center Database. All or part of the data used in the research were authorized by and received from MJ Health Research. Foundation (authorization code: AP_A2020019). Any interpretation or conclusion described in the paper does not represent the views of MJ Health Research Foundation. Due to legal restrictions imposed by the government of Taiwan concerning the “Personal Information Protection Act”, data cannot be made publicly available. Requests for data can be sent as a formal proposal to the MJ Health Examination Center Database (https://www.mjhrf.org/ (accessed on 15 March 2022)).
